# Knockdown of circFOXO3 ameliorates cigarette smoke-induced lung injury in mice

**DOI:** 10.1186/s12931-021-01883-w

**Published:** 2021-11-17

**Authors:** Lei Zhou, Bo Wu, Jun Yang, Bing Wang, Jing Pan, Donghui Xu, Chunling Du

**Affiliations:** Department of Respiratory Medicine, QingPu Branch of Zhongshan Hospital Affiliated To Fudan University, No.1158 Gongyuan Dong Road, Shanghai, 201700 China

**Keywords:** COPD, Circular RNA FOXO3, miR-214-3p, NF-κB, Lung inflammation

## Abstract

**Background:**

Chronic obstructive pulmonary disease (COPD) remains a prevalent chronic airway inflammatory disease. Circular RNAs (circRNAs) are associated with inflammation regulation; therefore, we examined distinct effects of circRNA FOXO3 (circFOXO3) against pneumonic inflammatory processes in COPD.

**Methods:**

We first quantified and localized circFOXO3 in mouse lung epithelial cell line MLE12 by quantitative reverse-transcription PCR and in situ hybridization. Next, circFOXO3 was suppressed by therapeutic administration of circFOXO3 knockdown lentivirus in mice exposed to air or cigarette smoke (CS) for 12 weeks, and several hallmarks of COPD were evaluated.

**Results:**

We noticed that circFOXO3 is upregulated in CS-exposed lungs and cigarette smoke extract (CSE)-treated murine alveolar epithelial cells. Knockdown of circFOXO3 attenuated the release of CXCL1 and IL-6 as well as inflammatory processes in the lungs of CS-exposed mice. In addition, we identified miR-214-3p as a circFOXO3-targeted microRNA. MiR-214-3p overexpression exerted protective effects against pneumonic inflammation after CS exposure. Silencing of circFOXO3 downregulated IKK-β mRNA (miR-214-3p’s target), resulting in the dysfunction of the NF-κB signaling pathway and attenuation of CSE-induced inflammatory-cytokine expression.

**Conclusions:**

Collectively, these findings reveal a crucial function of circFOXO3 in the pathological remodeling related to CS-induced inflammatory processes. Hence, circFOXO3 might be a good target for the treatment of inflammatory disorders similar to CS-induced lung inflammation.

## Background

Chronic obstructive pulmonary disease (COPD) remains an ever-growing health problem globally and is characterized by persistent lung inflammation, including systemic complications or comorbidities, causing chronic morbidity and eventually mortality [1]. To date, cigarette smoking has been the most fundamental risk factor of COPD. Even though smoking discontinuation without delay is the only practical remedy for COPD, it only slightly ameliorates the accelerated deterioration of lung function. Innovative therapeutic targets and elucidation of their mechanisms of action are urgently needed to develop efficacious therapies for COPD.

Circular RNA (circRNA) is a novel type of noncoding RNA with closed circular structure and is challenging to degrade; its expression is very tightly regulated [2, 3]. The latest studies confirmed that circRNA fragments are rich in microRNA (miRNA, miR)-binding sites and mimic the function of miRNA sponges within the cell, thereby reducing any inhibitory impact of miRNAs against their target genes and consequently boosting expression levels of these target genes [4]. Increasing evidence indicates that circRNAs perform crucial functions in cell development and engage in distinct pathological and physiological processes [5]. Notably, alterations of circRNA expression levels may correlate with the pathophysiology of individual diseases, e.g., pulmonary and airway diseases, such as COPD. Zeng and colleagues have performed RNA sequencing and found unusual circRNA expression patterns in central HSAECs exposed to a cigarette smoke extract (CSE). Certain dysregulated circRNAs may partake in the cellular airway response to cigarette smoke (CS)-provoked stress, thus offering various justifications for testing circRNA involvement in COPD [6]. Qiao et al. have demonstrated that a circ-RBMS1 knockdown mitigates CSE-induced apoptosis, oxidative stress, and lung inflammatory processes by upregulating FBXO11 or via miR-197-3p in 16HBE cells [7]. CircRNA FOXO3 (circFOXO3) is a conventional exonic circRNA, which has been shown to either increase or attenuate specific pathological changes in several human cancers [8, 9]. It has been reported that circFOXO3 can promote cardiac senescence, and a knockdown of circFOXO3 reduces cardiac ischemia–reperfusion injury [10, 11]. Despite circFOXO3’s practical importance, its possible participation in the regulation of CS-induced inflammatory responses is still poorly studied.

The present work revealed that circFOXO3 is significantly upregulated in CS-exposed lungs and CSE-treated murine alveolar epithelial cells. A knockdown of circFOXO3 reduced CS-induced pulmonary inflammation by downregulating IKK-β mRNA (the target of miR-214-3p), thereby resulting in the dysfunction of NF-κB signaling. Therefore, suppression of circFOXO3 may be an innovative preventive strategy against CS-induced inflammation.

## Methods

### Animal experiments

All laboratory methods requiring animal experiments were authorized by the Animal Care and Use Committee of QingPu Branch of Zhongshan Hospital Affiliated to Fudan University. C57BL/6 male adolescent mice aged 6 to 8 weeks were acquired from SLAC (Shanghai, China) and subdivided into control and experimental groups. Before treatment, the mice were allowed to get used to the new environment with normal access to water and rodent feed for 1 week. All the mice were then subjected to full-body CS exposure following previously described methods [12, 13]. Each group included six mice. The control group was not exposed to CS, but other mice were exposed to tobacco smoke from five cigarettes, two times a day, 5 days per week for 12 weeks. Mice in the control group were kept in ambient air only. For lentivirus transduction, a circFOXO3 knockdown lentivirus (shcircFOXO3), obtained from GenePharma (Shanghai, China) or an miR-214-3p agomir, and their corresponding negative controls (NCs) were injected into the tail vein of mice once every 2 weeks after the first CS exposure. The sequence of shcircFOXO3 was 5′-*CCGG*GGGCAAAGCAGAACTCCATTT*CTCGAG*AAATGGAGTTCTGCTTTGCCC*TTTTTG*-3′ [10]; it was provided by GenePharma.

### Bronchoalveolar lavage fluid (BALF)

The mice were euthanized with an overdose of pentobarbital 24 h after the last CS exposure. BALF collection was conducted as previously described [13]. Through a tracheal cannula, mouse lungs were lavaged with 400 μl of phosphate-buffered saline (PBS) three times. After that, all cells were counted in a Neubauer counting chamber, while some cell types (at least 400 cells each) were subjected to cytocentrifugation to determine their total cell number after lavaged Wright–Giemsa staining.

### Quantitative reverse-transcription PCR (qRT-PCR)

Total-RNA samples were isolated using the miRNeasy Mini Kit (Qiagen, Germany) (for small RNAs) or the TRIzol reagent (Invitrogen, Carlsbad, CA, USA) (for mRNA). Primers specific to first-strand cDNA were obtained using the PrimeScript™ Reverse Transcriptase Kit (TaKaRa, Japan). For miR-214-3p, stem-loop reverse transcription was conducted using the Moloney murine leukemia virus (M-MLV) Reverse Transcriptase Kit (Invitrogen). qRT-PCR was carried out with the SYBR Premix Ex Taq II Kit (TaKaRa) on a CFX Connect Real-Time System (Bio-Rad, Feldkirchen, Germany). All reactions were carried out in triplicate. *U6* and *GAPDH* were used as endogenous controls for miRNA and mRNA, respectively. Relative expression levels of RNAs were determined by the comparative Ct (also known as 2^−ΔΔCt^) method. The PCR primers for circFOXO3 were 5’-GTGGGGAACTTCACTGGTGCTAAG-3’ and 5’-GGGTTGATGATCCACCAAGAGCTCTT-3’. The PCR primers for CXCL1 were 5’-GGCTGGGATTCACCTCAA-3’ and 5’-GCGACCATTCTTGAGTGT-3’. The PCR primers for IL6 were 5’-ATGTTCTCTGGGAAATCGTGGAAAT-3’ and 5’-TCTCTGAAGGACTCTGGCTTTGT-3’. The PCR primers for IKK-β were 5’-CATCGGCTCTTAGATACCTT-3’ and 5’-ACTTCACTGCTCCATTCAA-3’. The PCR primers for GAPDH were 5’-CTTAGGTTCATCAGGTAAACTCAG-3’ and 5’-CATGTAGTTGAGGTCAATGAAGG-3’.

### Enzyme-linked immunosorbent assay (ELISA)

The levels of mouse CXCL1 and IL-6 in lung tissue homogenates or cell-free culture supernatants were quantified by means of ELISA kits from R&D system (Minnesota, USA) and Cloud-Clone Corporation (Wuhan, Hubei, China), respectively.

### Hematoxylin and eosin (H&E) staining

Lung parenchyma samples were fixed in 4% paraformaldehyde, embedded into paraffin, and were cut into sequential 4-μm-thick slices before staining with H&E for histological analysis by widely used standard procedures. The slices were observed under a fluorescence microscope at 200 × magnification (Nikon, Japan).

### Immunohistochemical (IHC) staining

The IHC staining protocol employed here is described in our previous study [14]. Lung parenchyma samples were fixed in 4% paraformaldehyde. Precisely 4-μm-thick three-dimensional paraffin block sections were prepared on a Leica slicer (Leica, Wetzlar, Germany). The tissue sections were heated at 60 °C for 2 h, deparaffinized with xylene, and then rehydrated. For epitope retrieval, the sections were kept in EDTA antigen retrieval buffer before microwaving. After that, all sections were treated with hydrogen peroxide/PBS for 30 min, supplemented with 1% bovine serum albumin, which blocks nonspecific binding. At 4 °C overnight, the tissue samples were incubated with primary antibodies (against CD11b and CD68; Abcam, Cambridge, UK), and then with secondary antibodies followed by 3,3′-diaminobenzidine (DAB) staining. The tissue sections were examined under a light microscope (Nikon), and images were quantified by means of the Image-Pro 6.0^+^ software (Media Cybernetics Corp., Silver Spring, USA).

### CSE preparation

CSE was made as described elsewhere [14]. Briefly, the smoke from four tobacco plants was bubbled through 30 mL of PBS. The extract was next purified by passing it through a 0.22 μm cigarette filter; this CSE was assumed to have 100% concentration. Before use, the pure extract was diluted to 2.5% concentration with a culture medium.

### Cell culture and treatment

MLE12 cells were acquired from the American Type Culture Collection (Manassas, VA, USA) and were cultured in the RPMI-1640 medium supplemented with 10% of FBS in a humidified atmosphere containing 5% of CO_2_ at 37 °C. The cells were exposed to 2.5% CSE for 24 h, after reaching 70–80% confluence. For knockdown or overexpression assays, the cells were either infected with the shcircFOXO3-expressing lentivirus or transfected with the miR-214-3p mimic or inhibitor (GenePharma). After 48 h, the cells were treated with 2.5% CSE for another 24 h, and then, they were pelleted by centrifugation for further experiments.

### Western blotting assay

After cell lysis, individual proteins were separated by sodium dodecyl sulfate polyacrylamide gel electrophoresis (SDS-PAGE) in a gel containing 10% of acrylamide. Trans-blotting onto a nitrocellulose membrane by electrophoresis was performed in 1 × Tris/glycine buffer containing 20% of methanol at 4 °C for 2 h. The membranes were blocked with TBST containing 5% of nonfat dry milk powder for 30 min and then were incubated overnight with the following primary antibodies: anti-IKK-β (Abcam), anti-phospho- (p-)IκBα (Cell Signaling Technology, Danvers, MA, USA), anti-p-p65 (Cell Signaling Technology), anti-p65 (Cell Signaling Technology), and anti-GAPDH (Proteintech, Chicago, IL, USA) at 4 °C. The membranes were washed (three times for 5 min) with TBST and then probed with secondary antibodies for 30 min. After rinsing (three times for 5 min), the bound antibodies were imaged by means of the ECL Detection Kit. The ImageJ software (NIH, Bethesda, MD, USA) was used to quantify intensity of protein bands.

### Immunofluorescence staining

Cells were plated on 15 mm cell slides (Nest Biotechnology Co. Ltd., Wuxi, China) within wells of 24-well plates, and 4% paraformaldehyde was applied to fix the cells. To permeabilize the cells, 0.5% Triton X-100 was employed. After blocking with 5% BSA for 30 min, the cells were first incubated at 4 °C with primary antibodies (against IKK-β and p65) overnight and then with a fluorescent (Alexa Fluor 555–conjugated) secondary antibody in the dark for 1 h. Fluorescence images were captured using a Nikon microscope.

### A luciferase reporter assay

CircFOXO3 and a fragment from the *IKK-β* 3′ untranslated region (3′UTR) carrying one predicted (either wild-type or mutant) miR-214-3p–binding site were synthesized and cloned into the pmirGLO dual-luciferase target expression vector (Promega Corp., Madison, WI, USA). Cells seeded in a 6-well plate were transfected with each pmirGLO vector expressing either a wild-type or mutated RNA and with the prepared miRNAs via the Lipofectamine 3000 reagent (Invitrogen). After 48 h, the cells were tested in a dual-luciferase reporter assay system (Promega Corp.).

### RNA-binding protein immunoprecipitation (RIP)

These assays were conducted using the Magna RIP™ RNA Binding Protein Immunoprecipitation Kit (Millipore, USA) according to the manufacturer’s instructions. Briefly, cell lysates were incubated with RIP immunoprecipitation buffer containing either AGO2-conjugated or IgG-conjugated magnetic beads. Coprecipitated RNAs were detected by qRT-PCR.

### Fluorescent in situ hybridization (FISH)

To detect circFOXO3 expression in cells, we utilized FISH with a mixture of DNA oligo probes labeled with Cy5, which were specific for either endogenous or ectopically expressed circFOXO3. A scrambled sequence labeled with Cy5 served as an NC. This assay was performed as described previously [10].

### Statistical analysis

Individually, experimental data of at least three independent experiments are presented as the mean ± SD. All data were evaluated for significance with Student’s *t* test. Statistical significance was set to *P* < 0.05.

## Results

### CircFOXO3 expression is increased in lung parenchyma of a mouse model and MLE12 cells after CS treatment

**Fig. 1:**
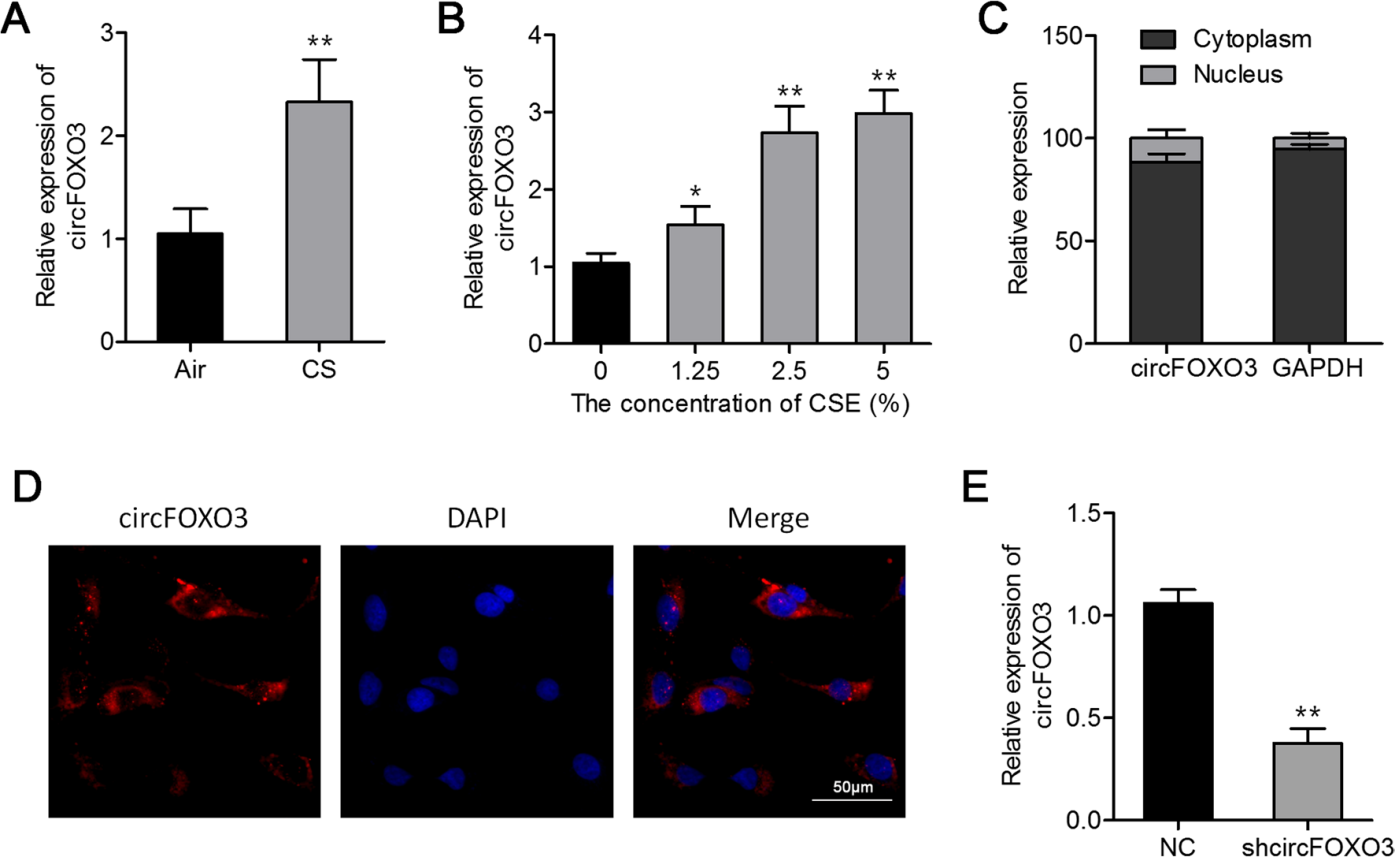
CS treatment increased the level of circFOXO3 in the lungs of model mice and in MLE12 cells. **A** The circFOXO3 expression levels in the lung parenchyma samples from CS-exposed and not CS-exposed mice (n = 6) were determined by qRT-PCR. **B** qRT-PCR analysis of the expression of circFOXO3 in MLE12 cells treated with various concentrations of CSE for 24 h. **C** The qRT-PCR data show high abundance of circFOXO3 in either the cytoplasm or nucleus of MLE12 cells. *GAPDH* mRNA served as an internal control for the cytoplasmic RNAs. **D** RNA FISH for circFOXO3. **E** qRT-PCR analysis of the circFOXO3 expression in MLE12 cells infected with the shcircFOXO3-expressing lentivirus. *P < 0.05, **P < 0.01

We first explored the effects of CS on circFOXO3 levels in mouse lungs. As shown in Fig. 1A, CS increased circFOXO3 expression in lung parenchyma in vivo. Similarly, we observed that CSE upregulated circFOXO3 in MLE12 cells in vitro (Fig. 1B). In addition, we performed qRT-PCR analysis of nuclear and cytoplasmic circFOXO3 and a FISH assay for circFOXO3, and the results showed that the circular type of FOXO3 RNA was preferentially localized in the cytoplasm of MLE12 cells (Fig. 1C, D). Then, we prepared a lentivirus expressing a short hairpin RNA (shRNA) against mouse circFOXO3 (shcircFOXO3). The infection with the lentivirus expressing shcircFOXO3 reduced levels of endogenous circFOXO3 in MLE12 cells (Fig. 1E).

### The knockdown of circFOXO3 suppresses inflammation in CS-exposed lungs in vivo*.*

**Fig. 2:**
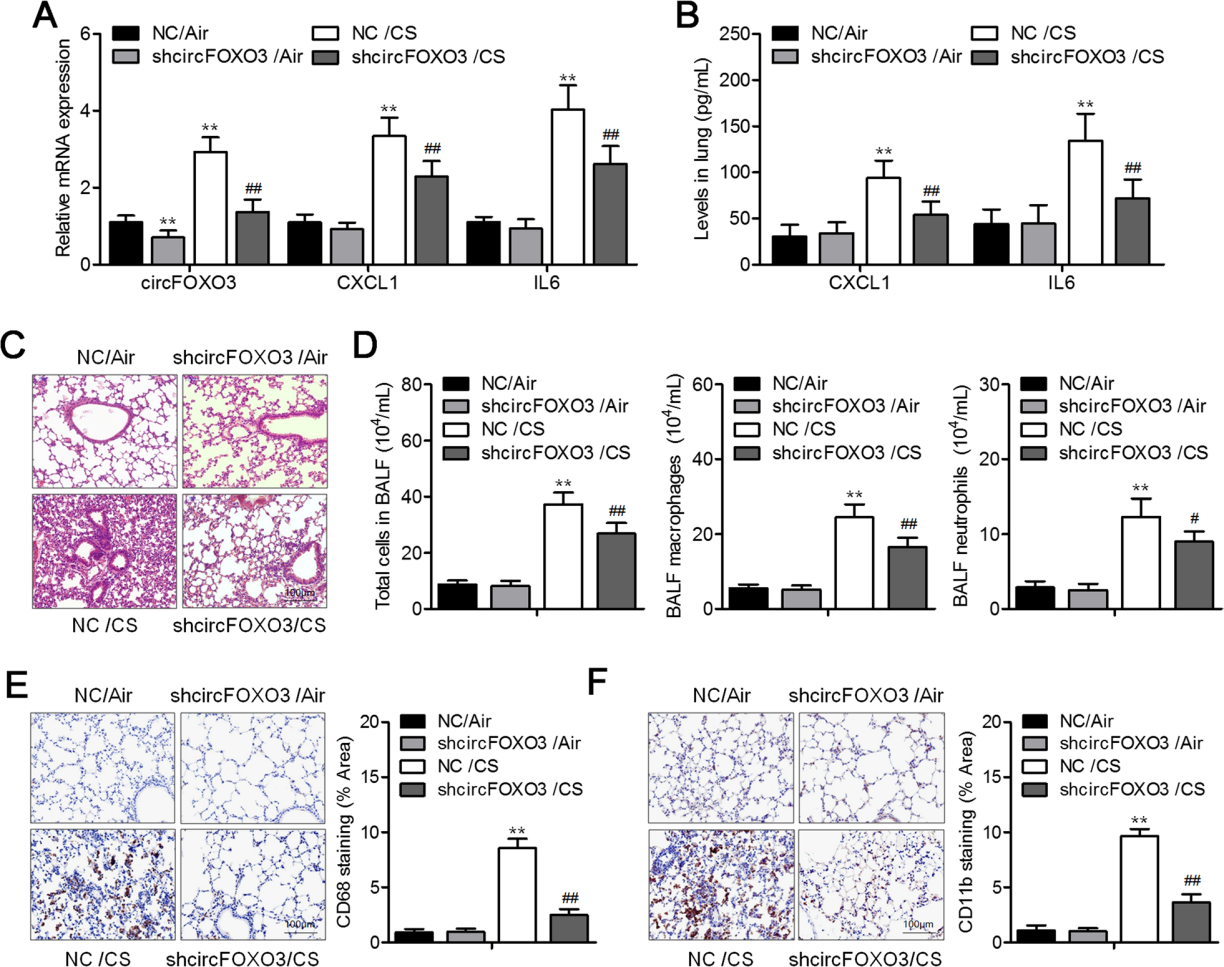
The knockdown of circFOXO3 prevented CS-induced inflammation in mice lungs. **A** qRT-PCR analysis of the levels of *CXCL1* and *IL-6* mRNA and circFOXO3 in the lung homogenates. **B** The inflammatory cytokines CXCL1 and IL-6 within the lung homogenates were quantified by ELISAs. **C** Illustrative micrographs of H&E-stained lung parenchyma samples. **D** The numbers of all cells, macrophages, and neutrophils in the BALF of mice. **E** The infiltration by macrophages was analyzed by CD68 staining in the lungs. **F** Representative photographs of immunostaining for CD11b show the infiltration by neutrophils in the lungs and quantitative analysis of the infiltration by CD11b^+^ cells (shown on the right). **P < 0.01 vs. negative shRNA control (NC)/Air group or shcircFOXO3/Air group, ^#^*P* < 0.05 and ^##^*P* < 0.01 vs. NC/CS group

To investigate the influence of circFOXO3 on CS-induced inflammatory processes, C57BL/6 mice were exposed or not exposed to CS (breathed laboratory air) for 12 weeks. The mice were injected with the circFOXO3 knockdown lentivirus via the tail vein once every 2 weeks. qRT-PCR analysis of the lung tissues showed that the administration of the circFOXO3 knockdown lentivirus decreased circFOXO3 levels (Fig. 2A). Furthermore, CS induced a significant increase in CXCL1 and IL-6 expression levels in the mouse lungs, and this effect was significantly attenuated by the circFOXO3 knockdown (Figs. 2A, B). Histopathological examination of such lungs after H&E staining revealed that the circFOXO3 knockdown weakened the infiltration by most of inflammatory cells in the alveolar spaces exposed to CS (Fig. 2C). Moreover, the circFOXO3 knockdown significantly attenuated the CS-induced increase in cumulative neutrophils, macrophages, and BALF cells (Fig. 2D). In agreement with these findings, IHC staining of lung CD11b and CD68 revealed that the circFOXO3 knockdown decreased the numbers of infiltrating neutrophils and macrophages (Figs. 2E, F).

### CircFOXO3 serves as the sponge of miR-214-3p thus raising IKK-β expression

**Fig. 3:**
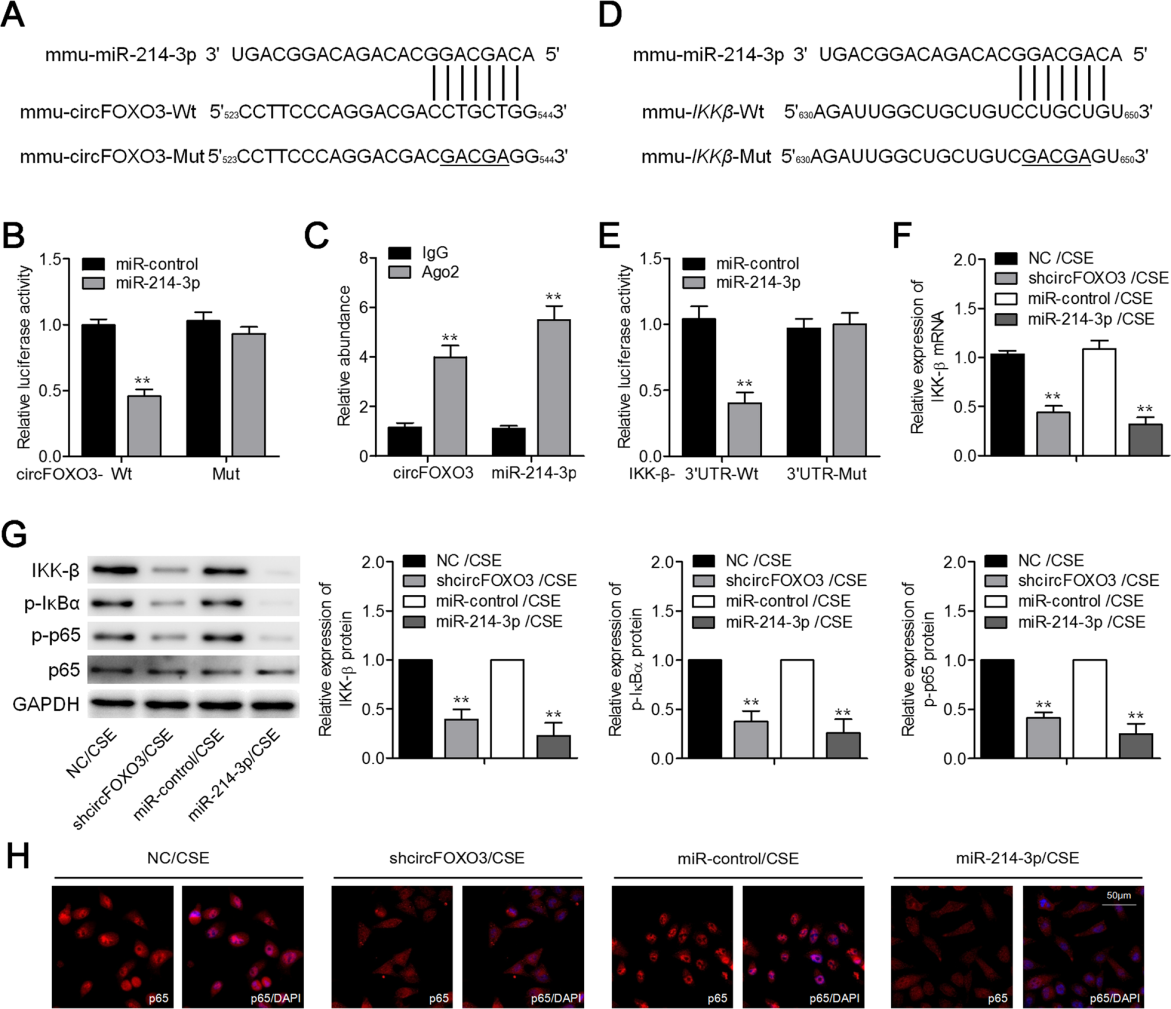
CircFOXO3 operates like a sponge of miR-214-3p, consequently raising IKK-β expression in MLE12 cells. **A** A natural miR-214-3p–binding site is predicted in circFOXO3. **B** MLE12 cells were cotransfected with either the wild-type or mutant circFOXO3 reporter plasmid and either the miR-214-3p mimic or miR-control mimic followed by a luciferase reporter assay. **C** A physical association of circFOXO3 with miR-214-3p and AGO2 was determined by the RIP assay in 2.5% CSE–treated MLE12 cells. **D** The prospective binding site for miR-214-3p predicted in *IKK-β* mRNA. **E** MLE12 cells were individually cotransfected with either the wild-type or mutant IKK-β 3'UTR reporter plasmid and either the miR-214-3p mimic or miR-control mimic followed by a luciferase reporter assay. **F** Expression levels of *IKK-β* mRNA in circFOXO3 knockdown MLE12 cells or miR-214-3p–overexpressing MLE12 cells after treatment with 2.5% CSE, according to qRT-PCR analyses. **G** Western blot analyses of protein levels of IKK-β, p-IκBα, p-p65, p65, and GAPDH in the indicated cell groups. **H** Some patterns of p65 translocation were examined by immunofluorescence microscopy in the indicated cell groups. **P < 0.01

Given that circRNAs act principally as miRNA sponges to disable an miRNA and swiftly improve a target gene’s expression, we next ran a search for the endogenous miRNAs binding to circFOXO3. Many candidate miRNAs were predicted in the miRanda software. We were particularly interested in miR-214-3p because of its critical role in inflammatory processes [15, 16]. As depicted in Fig. 3A, the circFOXO3 sequence has a representative miR-214-3p–binding site. To verify whether this presumed miR-214-3p–binding site works as expected, a luciferase assay was performed. The results confirmed that transfection of miR-214-3p can reduce Luc-circFOXO3 activities, although this transfection did not appear to influence Luc-circFOXO3-mut activities in MLE12 cells (Fig. 3B). RIP assay results suggested that circFOXO3 and miR-214-3p were both enriched within the precipitate following immunoprecipitation with the anti-AGO2 antibody in contrast to the IgG control, thereby further corroborating the direct binding between circFOXO3 and miR-214-3p (Fig. 3C). Furthermore, given that *IKK-β* mRNA has been reported as a target of miR-214-3p [16], an appropriate luciferase assay was carried out, and its outcome indicated that miR-214-3p overexpression diminished Luc-IKK-β-3'UTR activities but did not reduce Luc-IKK-β-3'UTR-mut activities in MLE12 cells (Fig. 3D, E). We then examined the expression of IKK-β and found that the knockdown of circFOXO3 or transfection of the miR-214-3p mimic significantly decreased mRNA and relative protein expression levels of IKK-β in CSE-treated MLE12 cells (Fig. 3F, G). When IKK-β remains a functional protein, it phosphorylates IκBα, triggering its degradation and launches the NF-κB signaling pathway. Next, we determined the phosphorylation of IκBα and p65 (NF-κB subunit) in MLE12 cells. Western blotting analysis suggested that the knockdown of circFOXO3 or miR-214-3p overexpression dramatically decreased p-IκBα and p-p65 levels in CSE-treated MLE12 cells (Fig. 3G). Of note, immunofluorescence assays uncovered diminished nuclear translocation of p65 after the knockdown of circFOXO3 or miR-214-3p overexpression in CSE-treated MLE12 cells (Fig. 3H).

### MiR-214-3p overexpression weakens CS-induced lung inflammation in vivo

**Fig. 4:**
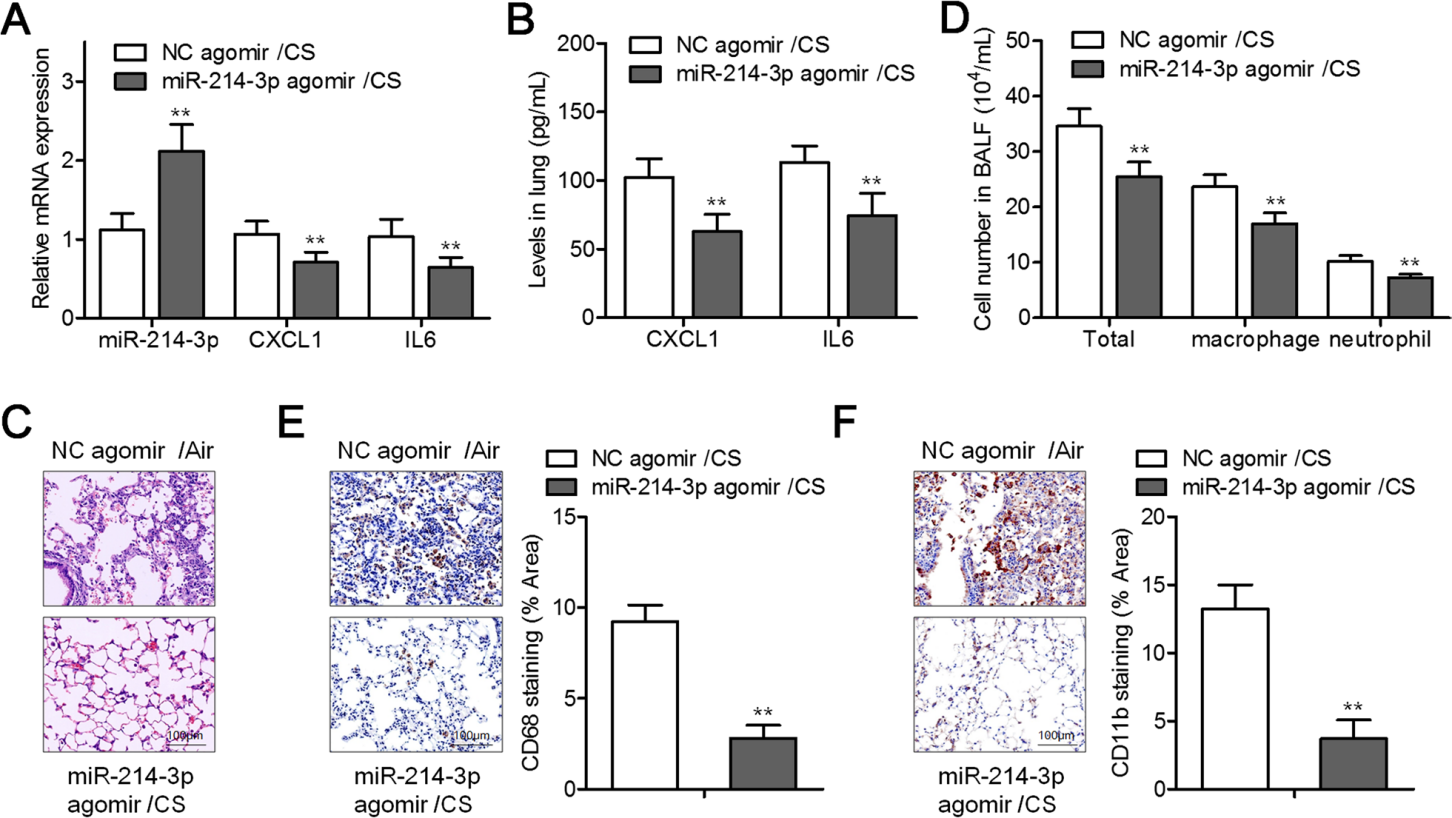
MiR-214-3p overexpression suppressed CS-induced lung inflammatory processes in vivo. **A** A qRT-PCR analysis of expression levels of *CXCL1* and *IL-6* mRNAs and miR-214-3p in the lung homogenates of CS-exposed mice treated with either the NC or miR-214-3p agomir. **B** The inflammatory cytokines CXCL1 and IL-6 in the lung homogenates, according to ELISAs. **C** Typical images of the H&E-stained lung parenchyma samples. **D** The separate counts of neutrophils, macrophages, and total cells within mouse BALF. **E** The infiltration by macrophages was analyzed by CD68 staining in the lungs. **F** Representative images of immunostaining for CD11b uncovered the infiltration of the lungs by neutrophils; quantitative analysis of the infiltration by CD11b^+^ cells (as presented on the right). **P < 0.01

We also studied the effects of miR-214-3p on CS-induced inflammation in vivo. Mice exposed to CS were injected with the miR-214-3p agomir (or NC agomir) via the tail vein. The qRT-PCR results indicated that miR-214-3p overexpression weakened the effects of CS on the expression of CXCL1 and IL-6 in the mouse lungs (Fig. 4A, B). H&E staining further confirmed that miR-214-3p overexpression attenuated the inflammatory-cell infiltration into alveolar space as compared with the group “CS exposure alone” (Fig. 4C). Similarly, miR-214-3p overexpression significantly attenuated the CS-induced increase in the numbers of neutrophils, macrophages, and total BALF cells (Fig. 4D). IHC staining of CD68 and CD11b in the lung sections showed that miR-214-3p overexpression diminished the numbers of infiltrating macrophages and neutrophils (Fig. 4E, F).

### CircFOXO3 enhances CSE-induced inflammatory-cytokine expression in MLE12 cells by upregulating the output of the miR-214-3p–IKK-β axis

**Fig. 5:**
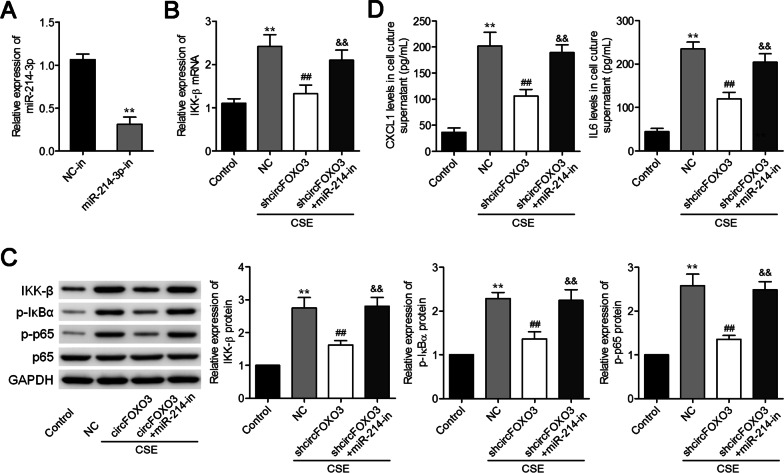
The miR-214-3p inhibitor attenuates the effects of circFOXO3 silencing thus upregulating IKK-β. **A** The effectiveness of miR-214-3p inhibition in MLE12 cells, judging from qRT-PCR data. **B** qRT-PCR analysis of *IKK-β* expression levels in circFOXO3 knockdown cells transfected with the miR-214-3p inhibitor followed by CSE treatment. **C** Western blotting analysis of the protein levels of IKK-β, p-IκBα, p-p65, p65, and GAPDH in the indicated cell groups. **D** Levels of CXCL1 and IL-6 in the culture supernatant were assessed by ELISAs. **P < 0.01 vs. Control group, ^##^*P* < 0.01 *vs*. NC/CSE group, ^&&^*P* < 0.01 *vs*. circFOXO3/CSE group

Finally, the miR-214-3p inhibitor was applied to investigate whether the effects of circFOXO3 silencing on CSE-induced inflammatory cytokine expression might be reversed by an miR-214-3p knockdown in MLE12 cells. After the circFOXO3 silencing, the miR-214-3p inhibitor significantly reversed the downregulation of IKK-β mRNA and protein expression (Fig. 5A–C). In addition, the miR-214-3p inhibitor attenuated the p-IκBα and p-p65 downregulation caused by the silencing of circFOXO3 (Fig. 5C). Moreover, the miR-214-3p inhibitor reversed the circFOXO3 silencing–mediated suppression of CXCL1 and IL-6 expression levels in MLE12 cells after CSE treatment (Fig. 5D). These data convincingly indicated that circFOXO3 enhances CSE-induced expression of inflammatory cytokines in MLE12 cells as an miR-214-3p sponge thereby weakening the effects of miR-214-3p in the circFOXO3–miR-214-3p–IKK-β axis.

## Discussion

The roles of circRNAs in the progression of inflammation-related diseases has attracted much attention recently [17, 18]. Our study was focused on the function and mechanisms of action of increased circFOXO3 expression in COPD progression. We demonstrated that a knockdown of circFOXO3 attenuates CS-induced inflammation via suppression of miR-214-3p–IKK-β axis output. These observations point to intrinsic functions of circFOXO3 in the pathogenesis of pulmonary inflammation in COPD.

CircFOXO3 is an exonic circRNA that is widely expressed in the lungs, heart, and intestinal tissues [10]. It has been reported that circFOXO3 is overexpressed in the heart of mature mice and patients and is associated with unrestricted senescence, and that ectopic expression of circFOXO3 induced senescence and exacerbates doxorubicin-induced cardiomyopathy [10]. In addition, circFOXO3 is significantly upregulated in an ischemia–reperfusion–injured heart and hypoxia/reoxygenation–treated cardiomyocytes. Furthermore, a circFOXO3 knockdown can overcome ischemia–reperfusion pain and improve heart graft function [11]. It is also known that circFOXO3 is significantly upregulated after glutamate-induced oxidative stress in HT22 cells, and that circFOXO3 contributes to mitochondria-mediated apoptosis [19]. Here, we demonstrated that circFOXO3 tends to be upregulated in CS-exposed lungs and CSE-treated MLE12 cells. Functional analysis revealed that the knockdown of circFOXO3 attenuates the release of CXCL1 and IL-6 in the lungs of CS-exposed mice and the CS-induced upregulation of inflammatory cells, such as macrophages and neutrophils, in BALF. Consistently with these findings, striking decreases in both CD11b and CD68 levels were registered in circFOXO3 knockdown lungs after CS treatment as compared with the lungs after CS treatment alone. These results suggest that circFOXO3 inhibition can prevent CS-induced pulmonary inflammation.

CircRNAs can indirectly increase mRNA expression levels by sponging miRNAs [4]. To determine the mechanism of action of circFOXO3, we searched for potential target miRNAs and identified miR-214-3p. Earlier studies indicate that miR-214-3p performs a vital function in a variety of pathological processes in inflammatory conditions. For example, by transfecting miR-214-3p, Lei et al. have revealed its protective effects in extracellular vesicles from mesenchymal stem cells against radiation-induced lung injury [15]. Cao et al. have found that decreased miR-214-3p expression aggravates osteoarthritis progression and activates the NF-κB pathway [16]. In contrast, Yan et al. recently reported that miR-214-3p can intensify the kidney damage and inflammation triggered by hyperlipidemic pancreatitis caused by severe renal injury [20]. In the present study, we demonstrated a flexible relation between circFOXO3 and miR-214-3p as follows: (1) A luciferase reporter assay confirmed the direct binding of the predicted miRNA (miR-214-3p) to a binding site in circFOXO3; (2) RIP assays revealed that circFOXO3 and miR-214-3p are present together in a rele(vant RNA-induced silencing complex; (3) miR-214-3p overexpression suppressed CS-induced lung inflammation; (4) circFOXO3 was found to serve as an miR-214-3p sponge, consequently enhancing IKK-β expression and activating the NF-κB pathway. Thus, we provided evidence of a novel competitive-endogenous-RNA regulatory network in which circFOXO3 as a sponge interacts with miR-214-3p.

## Conclusion

In brief, our data show that the knockdown of circFOXO3 exerts a protective action against CS-induced inflammation, and that this action is mediated by downregulation of circFOXO3–miR-214-3p–IKK-β axis output and suppression of the NF-κB pathway. Therefore, the suppression of circFOXO3 may become a feasible therapeutic approach to CS-induced inflammatory disorders.

## Data Availability

All data generated and analyzed during the study are included in the published article and can be shared upon request.

## Abbreviations

COPD: Chronic obstructive pulmonary disease
CircRNAs: Circular RNAs
CircFOXO3: CircRNA FOXO3
MiRNA: MicroRNA
CS: Cigarette smoke
CSE: Cigarette smoke extract
BALF: Bronchoalveolar lavage fluid
qRT-PCR: Quantitative reverse-transcription PCR
ELISA: Enzyme-linked immunosorbent assay
RIP: RNA-binding protein immunoprecipitation
FISH: Fluorescent in situ hybridization

## References

[1] Wang Y, Liu J, Zhou JS, Huang HQ, Li ZY, Xu XC (2018). MTOR suppresses cigarette smoke-induced epithelial cell death and airway inflammation in chronic obstructive pulmonary disease. J Immunol.

[2] Han D, Li J, Wang H, Su X, Hou J, Gu Y (2017). Circular RNA circMTO1 acts as the sponge of microRNA-9 to suppress hepatocellular carcinoma progression. Hepatology.

[3] Ouyang Z, Tan T, Zhang X, Wan J, Zhou Y, Jiang G (2019). CircRNA hsa_circ_0074834 promotes the osteogenesis-angiogenesis coupling process in bone mesenchymal stem cells (BMSCs) by acting as a ceRNA for miR-942-5p. Cell Death Dis.

[4] Cortes-Lopez M, Miura P (2016). Emerging functions of circular RNAs. Yale J Biol Med.

[5] Lopez-Jimenez E, Andres-Leon E (2021). The implications of ncRNAs in the development of human diseases. ncRNA.

[6] Zeng N, Wang T, Chen M, Yuan Z, Qin J, Wu Y (2019). Cigarette smoke extract alters genome-wide profiles of circular RNAs and mRNAs in primary human small airway epithelial cells. J Cell Mol Med.

[7] Qiao D, Hu C, Li Q, Fan J (2021). Circ-RBMS1 knockdown alleviates CSE-induced apoptosis, inflammation and oxidative stress via up-regulating FBXO11 through miR-197-3p in 16HBE cells. Int J Chron Obstruct Pulmon Dis.

[8] Zhang S, Liao K, Miao Z, Wang Q, Miao Y, Guo Z (2019). CircFOXO3 promotes glioblastoma progression by acting as a competing endogenous RNA for NFAT5. Neuro Oncol.

[9] Shen Z, Zhou L, Zhang C, Xu J (2020). Reduction of circular RNA Foxo3 promotes prostate cancer progression and chemoresistance to docetaxel. Cancer Lett.

[10] Du WW, Yang W, Chen Y, Wu ZK, Foster FS, Yang Z (2017). Foxo3 circular RNA promotes cardiac senescence by modulating multiple factors associated with stress and senescence responses. Eur Heart J.

[11] Su Y, Zhu C, Wang B, Zheng H, McAlister V, Lacefield JC (2020). Circular RNA Foxo3 in cardiac ischemia-reperfusion injury in heart transplantation: a new regulator and target. Am J Transplant.

[12] D'Hulst AI, Vermaelen KY, Brusselle GG, Joos GF, Pauwels RA (2005). Time course of cigarette smoke-induced pulmonary inflammation in mice. Eur Respir J.

[13] Zhou H, Hua W, Jin Y, Zhang C, Che L, Xia L (2015). Tc17 cells are associated with cigarette smoke-induced lung inflammation and emphysema. Respirology.

[14] Tao Y, Sun Y, Wu B, Xu D, Yang J, Gu L, Du C (2021). Overexpression of FOXA2 attenuates cigarette smoke-induced cellular senescence and lung inflammation through inhibition of the p38 and Erk1/2 MAPK pathways. Int Immunopharmacol.

[15] Lei X, He N, Zhu L, Zhou M, Zhang K, Wang C (2020). Mesenchymal stem cell-derived extracellular vesicles attenuate radiation-induced lung injury via miRNA-214–3p. Antioxid Redox Signal.

[16] Cao Y, Tang S, Nie X, Zhou Z, Ruan G, Han W (2021). Decreased miR-214–3p activates NF-kappaB pathway and aggravates osteoarthritis progression. EBioMedicine.

[17] Li M, Hua Q, Shao Y, Zeng H, Liu Y, Diao Q (2020). Circular RNA circBbs9 promotes PM2.5-induced lung inflammation in mice via NLRP3 inflammasome activation. Environ Int.

[18] Saaoud F, Drummer IVC, Shao Y, Sun Y, Lu Y, Xu K (2021). Circular RNAs are a novel type of non-coding RNAs in ROS regulation, cardiovascular metabolic inflammations and cancers. Pharmacol Ther.

[19] Lin SP, Hu J, Wei JX, Ye S, Bu J, Xu W (2020). Silencing of circFoxO3 Protects HT22 cells from glutamate-induced oxidative injury via regulating the mitochondrial apoptosis pathway. Cell Mol Neurobiol.

[20] Yan Z, Zang B, Gong X, Ren J, Wang R (2020). MiR-214–3p exacerbates kidney damages and inflammation induced by hyperlipidemic pancreatitis complicated with acute renal injury. Life Sci.

